# Age- and gender-specific population attributable risks of metabolic disorders on all-cause and cardiovascular mortality in Taiwan

**DOI:** 10.1186/1471-2458-12-111

**Published:** 2012-02-10

**Authors:** Wuan-Szu Wang, Mark L Wahlqvist, Chih-Cheng Hsu, Hsing-Yi Chang, Wan-Chi Chang, Chu-Chih Chen

**Affiliations:** 1 Division of Biostatistics and Bioinformatics, Institute of Population Health Sciences, National Health Research Institutes, No. 35, Keyan Road, Zhunan, Miaoli County 35053, Taiwan; 2 Division of Health Service Research and Preventive Medicine, Institute of Population Health Sciences, National Health Research Institutes, No. 35, Keyan Road, Zhunan, Miaoli County 35053, Taiwan

## Abstract

**Background:**

The extent of attributable risks of metabolic syndrome (MetS) and its components on mortality remains unclear, especially with respect to age and gender. We aimed to assess the age- and gender-specific population attributable risks (PARs) for cardiovascular disease (CVD)-related mortality and all-cause mortality for public health planning.

**Methods:**

A total of 2,092 men and 2,197 women 30 years of age and older, who were included in the 2002 Taiwan Survey of Hypertension, Hyperglycemia, and Hyperlipidemia (TwSHHH), were linked to national death certificates acquired through December 31, 2009. Cox proportional hazard models were used to calculate adjusted hazard ratios and PARs for mortality, with a median follow-up of 7.7 years.

**Results:**

The respective PAR percentages of MetS for all-cause and CVD-related mortality were 11.6 and 39.2 in men, respectively, and 18.6 and 44.4 in women, respectively. Central obesity had the highest PAR for CVD mortality in women (57.5%), whereas arterial hypertension had the highest PAR in men (57.5%). For all-cause mortality, younger men and post-menopausal women had higher PARs related to Mets and its components; for CVD mortality, post-menopausal women had higher overall PARs than their pre-menopausal counterparts.

**Conclusions:**

MetS has a limited application to the PAR for all-cause mortality, especially in men; its PAR for CVD mortality is more evident. For CVD mortality, MetS components have higher PARs than MetS itself, especially hypertension in men and waist circumference in post-menopausal women. In addition, PARs for diabetes mellitus and low HDL-cholesterol may exceed 20%. We suggest differential control of risk factors in different subpopulation as a strategy to prevent CVD-related mortality.

## Background

Metabolic disorders such as arterial hypertension (AH), hypercholesterolemia, diabetes mellitus (DM), high triglycerides (TG), abdominal obesity, and metabolic syndrome (MetS) are associated with cardiovascular disease (CVD) and CVD-related mortality. Furthermore, metabolic disorders and MetS have contributed to all-cause mortality in U.S., European, Australian, and Asian populations [1-8]. Given the global public health burden of metabolic disorders, the corresponding metabolic risk factors warrant more careful examination to develop prevention strategies for various subpopulations.

The prevalence of MetS and metabolic disorders, as well as their associations with morbidity and mortality, vary according to gender, age, and ethnicity [3,8-12]. The age-related increase in CVD in women tends to be delayed compared to men and is often delayed until the post-menopausal period. These differences lead to differences in the risk factor associations for the gender-age interaction [11,13,14]. Although the prevalence of MetS increases with age, its association with mortality in older adults is not as significant as in the middle-aged population [10,15].

Significant associations between metabolic disorders and MetS and CVD mortality, as well as all-cause mortality, have been demonstrated in Asian populations [5,6,8]. However, relatively few studies have reported the corresponding population attributable risks (PARs) of these factors [5]. From a preventive perspective, PARs are a helpful measurement for planning public health interventions and creating new public policy. As such, it should be recalculated periodically [4,16], especially for individual risk factors in different subgroups. The aim of this study was to assess the age and gender effects of MetS and metabolic disorders, including high waist circumference (WC), AH, DM, high TG, and low high-density lipoprotein cholesterol (HDL-C) on all-cause and CVD-related mortality within an 8-year follow-up period.

## Methods

### Study population and mortality data

The study subjects were identified from the 2002 Taiwan Survey of Hypertension, Hyperglycemia, and Hyperlipidemia (2002 TwSHHH) [17-20], a cross-sectional study that followed the 2001 National Health Interview Survey (NHIS) of Taiwan. The 2001 NHIS was an island-wide health survey. Non-institutionalized households were selected through a multistage, stratified, systematic sampling design, with the numbers of sampling units proportional to the background population of each stratum (e.g., an administration district such as a county or a city). Half of the primary sampling units of the 2001 NHIS were selected, and all household members older than 15 years were interviewed for the 2002 TwSHHH study. Residents in military housing communities, medical facilities, schools, job training centers, dormitories, prisons, or offshore islands were excluded from the study. The subjects were linked to the National Death Registry, which includes mortality data from March 3, 2002 to December 31, 2009 and lists causes of death as defined in accordance with the International Classification of Diseases, ninth revision (ICD-9) [21]. A total of 2,092 men and 2,197 women aged 30 years and older were evaluated for associations between obesity-related metabolic risk factors and mortality during an 8-year follow-up period. There were 74 deaths in total. These deaths had ICD-9 codes of 250 (17 cases of DM, 23%); 390-398 (1 case of acute rheumatic fever and chronic rheumatic heart disease, 1.4%); 401 (5 cases of essential hypertension, 6.8%); 402 (1 cases of hypertensive heart disease, 1.4%); 410-414 and 429.2 (12 cases of ischemic heart diseases and unspecified CVD, 16.2%); 415-417, 424-427, and 429 (7 cases of diseases of the pulmonary circulation and all other forms of heart diseases, 9.5%); 428 (2 cases of heart failure, 2.7%); 430-438 (27 cases of cerebrovascular diseases, 36.5%); 440 (1 case of atherosclerosis, 1.4%); and 441 (1 case of aortic aneurysm and dissection, 1.4%). Participants with no death registry record were assumed to be alive for the entire study period. The median length of follow-up was 7.7 years. Because accidental deaths, such as motor vehicle collisions, suicide, and homicide, were unrelated to MetS, these deaths were excluded from the analyses. The Institutional Review Board of the National Health Research Institutes of Taiwan approved this study.

### Measurements and definition of obesity-related metabolic disorders

The results of the anthropometric measurements, blood pressure measurements, and blood sample analyses have been reported previously [17]. Central obesity was defined as a WC ≥ 90 cm in men and ≥ 80 cm in women. AH was defined as an average systolic blood pressure (SBP) of ≥ 140 mmHg, a diastolic blood pressure (DBP) of ≥ 90 mmHg, or treatment for previously diagnosed hypertension. DM was defined as a fasting plasma glucose (FPG) concentration ≥ 126 mg/dL (7 mmol/L) or the use of insulin or other hypoglycemic agents. High TG was defined as a serum triacylglycerol concentration ≥ 200 mg/dL (2.26 mmol/L). Low HDL-C was defined as a HDL-C < 40 mg/dL (1.03 mmol/L) in men and < 50 mg/dL (1.29 mmol/L) in women. MetS was defined using the National Cholesterol Education Program Adult Treatment Panel III (NCEP ATP III) clinical guideline (modified for Asian populations), which require the presence of at least three characteristics: 1. WC ≥ 90 cm in men and ≥ 80 cm in women (21); 2. SBP ≥ 130 mmHg, DBP ≥ 85 mmHg, or self-reported treatment with antihypertensive medications; 3. FPG ≥ 110 mg/dL (6.1 mmol/L) or the use of insulin or hypoglycemic agents; 4. HDL-C < 40 mg/dL (1.03 mmol/L) in men and < 50 mg/dL (1.29 mmol/L) in women; and 5. serum TG ≥ 150 mg/dL (1.7 mmol/L) [22]. Instead of using the definition from the International Diabetes Federation Chinese, which was developed for diabetes, we adopted the modified MetS definition from ATP III, which was officially recommended by Taiwan's Department of Health (DOH) [23].

### Statistical analysis

A Cox proportional hazard model was fitted using the SAS procedure "PROC PHREG" (SAS Institute, Cary, NC, USA) to estimate the mortality hazard ratios (HRs) predicted by the individual metabolic disorders and MetS, using data analyzed through December 31, 2009, the last day of follow-up. Adjusted HRs were obtained by accounting for potential confounding variables: age (30-45, 45-55, and ≥ 55 years old), coronary artery disease (CAD), stroke, BMI (< 18.5, 18.5-24, 24-27, and > 27), personal behaviors (smoking, alcohol drinking, betel nut chewing, and exercising), and socioeconomic status (income). CAD and stroke were defined as having a self-reported history of CAD or stroke before the survey. The BMI cutoffs were adopted by Taiwan's DOH for obesity classification in a Taiwanese Population [24] instead of the cutoffs recommended by the World Health Organization (WHO). Smoking was defined as ever having smoked for more than 100 cigarettes. Alcohol drinking, betel nut chewing, and exercise were defined as currently having the respective behavior. Socioeconomic status was classified as no income, low income (less than 10,000 New Taiwan Dollars [NTDs]), secondary low income (10,000 to 40,000 NTDs), and medium income (greater than 40,000 NTDs). Pearson Chi-squared statistics were employed to test for gender differences. A Kaplan-Meier analysis (SAS procedure "PROC LIFETEST") was used to estimate the cumulative probability of the survival curves for all-cause and CVD-related mortality. Log-rank tests were used to test for subgroup differences. The significance level was set to *p *< 0.05.

To assess the contribution of the individual metabolic risk factors, PARs were calculated using the following equation [16]:

(1) PAR=pd(HR-1)/HR

where *pd *is the proportion of disease-specific exposure to the given risk factor among total deaths, and *HR *is the adjusted hazard ratio obtained from the Cox regression model. To avoid unstable estimates due to a low mortality rate, the bootstrap method was employed to generate 95% confidence intervals (CI) for the PARs by taking the 2.5 and 97.5 percentiles among 1000 re-sampled datasets using the SAS procedure "PROC SURVEYSELECT". Subgroup analyses for the HRs and PARs of the metabolic risk factors for all-cause and CVD-related mortalities were performed for women and men separately. In addition to stratifying the groups by gender, the subjects were further divided by age (30-45, 45-55, and ≥ 55 years old). Additionally, women were stratified by menopausal status (45-55, and ≥ 55 years) and analyzed separately from men.

## Results

### Baseline characteristics

Table 1 summarizes the baseline characteristics and prevalence of obesity-related metabolic disorders of the 2,092 men and 2,197 women who participated in the study. The numbers and percentages of all-cause and CVD-related deaths for each variable category are also listed. There were significant gender differences in age, BMI, alcohol drinking, smoking, betel nut chewing, exercise, education level, marital status, and income (p < 0.05). Men were substantially more likely to drink alcohol, smoke, and chew betel nuts than women. The prevalence of MetS, AH, DM, and elevated TG was also higher in men.

**Table 1:** Demographic characteristics and prevalence of metabolic disorders

Characteristics (%)	Men (n = 2092)	Death		Women (n = 2197)	Death		p-value^c^
		**All-cause (n = 167)**	**CVD (n = 42)**		**All-cause (n = 91)**	**CVD (n = 32)**	

Age (yrs.)							0.0001

30-45	920 (44.0)	16 (9.6)	6 (14.3)	1054 (48.0)	13 (14.3)	1 (3.1)	
45-55	533 (25.5)	28 (16.8)	5 (11.9)	598 (27.2)	15 (16.5)	3 (9.4)	
≥ 55	639 (30.5)	123 (73.7)	31 (73.8)	545 (24.8)	63 (69.2)	28 (87.5)	

BMI^a ^(kg/m²)							< 0.0001

< 18.5	56 (2.7)	10 (6.0)	1 (2.7)	125 (5.7)	9 (9.9)	3 (10.3)	
18.5-24.9	1273 (60.9)	107 (64.1)	25 (67.6)	1500 (68.3)	48 (52.8)	18 (62.1)	
25-29.9 ≥	679 (32.5)	44 (26.4)	9 (24.3)	479 (21.8)	28 (30.8)	8 (27.6)	
30	84 (4.0)	6 (3.6)	2 (5.4)	93 (4.2)	6 (6.6)	0 (0.0)	

BMI^b ^(kg/m²)							< 0.0001

< 18.5	56 (2.7)9	10 (6.0)	1 (2.4)	125 (5.7)	9 (9.9)	3 (9.4)	
18.5-24	78 (46.8)	84 (50.3)	19 (45.2)	1279 (58.2)	39 (42.9)	16 (50.0)	
24-27	708 (33.8)	57 (34.1)	15 (35.7)	528 (24.0)	25 (27.5)	9 (28.1)	
≥ 27	350 (16.7)	16 (9.6)	7 (16.7)	265 (12.1)	18 (19.8)	4 (12.5)	

Alcohol drinking	982 (46.9)	65 (38.9)	13 (31.0)	258 (11.7)	10 (11.0)	3 (9.4)	< 0.0001

Smoking	1116 (53.4)	104 (62.3)	24 (57.1)	85 (3.9)	9 (9.9)	3 (9.4)	< 0.0001

Betel nut chewing	660 (31.6)	57 (34.1)	16 (38.1)	61 (2.8)	5 (5.5)	2 (6.3)	< 0.0001

Excercise	1076 (51.4)	90 (53.9)	23 (54.8)	1204 (54.8)	42 (46.2)	12 (37.5)	0.03

Education							< 0.0001

Less than elementary school	550 (26.3)	94 (56.3)53 (31.7) 20 (12.0)	24 (57.1)13 (31.0)5 (11.9)	811 (37.0)996 (45.4)388 (17.7)	69 (75.8)19 (20.9) 3 (3.3)	27 (84.4) 5 (15.6) 0 (0.0)	
High school diploma	996 (47.6)						
More than high school	546 (26.1)						

Marital status							< 0.0001

Married	1804 (86.3)	133 (79.6)	34 (81.0)1 (2.4)3 (7.1)4 (9.5)	1780 (81.0) 87 (4.0) 216 (9.8) 114 (5.2)	49 (53.9) 4 (4.4) 37 (40.7) 1 (1.1)	15 (46.9) 1 (3.1)16 (50.0) 0 (0.0)	
Divorced or separated	54 (2.5)	4 (2.4)					
Widower or widow/Living together Single	60 (2.8) 174 (8.4)	16 (9.6) 14 (8.4)					

Income							< 0.0001

No income	228 (10.9)	44 (26.5)	10 (23.8)	760 (34.7)	47 (51.7)	19 (59.4)	
Less than 10,000 NTD	197 (9.5)	33 (19.9)	9 (21.4)	263 (12.0)	27 (29.7)	12 (37.5)	
10,000-40,000 NTD	888 (42.6)	63 (38.0)	18 (42.9)	886 (40.5)	14 (15.4)	1 (3.1)	
More than 40,000 NTD	772 (37.0)	26 (15.7)	5 (11.9)	281 (12.8)	3 (3.3)	0 (0.0)	

Self-reported CVD	277 (13.3)	47 (28.1)	19 (45.2)	290 (13.2)	36 (40.0)	18 (58.1)	0.9667

MetS	467 (22.3)	58 (34.7)	24 (57.1)	347 (15.8)	39 (42.9)	20 (62.5)	< 0.0001

High WC	702 (33.6)	64 (38.3)	21 (50.0)	742 (33.8)	56 (61.5)	25 (78.1)	0.88

AH	596 (28.5)	94 (56.3)	32 (76.2)	422 (19.2)	50 (55.0)	20 (62.5)	< 0.0001

DM	188 (9.0)	35 (21.0)	16 (38.1)	147 (6.7)	22 (24.2)	11 (34.4)	

HighTG	464 (22.2)	35 (21.0)	13 (31.0)	241 (11.0)	20 (22.0)	11 (34.4)	< 0.0001

Low HDL-C	479 (22.9)	50 (29.9)	15 (35.7)	522 (23.8)	25 (27.5)	17 (53.1)	0.50

a: BMI classification according to the recommended standard of the WHO

b: BMI classification according to the recommended standard of the Taiwan

Department of Health

c: Significance level of two sample *t*-test or chi-squared test for the difference in gender

^1^BMI, body mass index; DM, diabetes mellitus; AH, arterial hypertension; HDL-C, high-density lipoprotein cholesterol; MetS, metabolic syndrome; NTD, New Taiwan Dollar; TG, triglycerides; WC, waist circumference

^2^Central obesity was defined as WC ≥ 90 cm in men and ≥ 80 cm in women; AH was defined as an average SBP of ≥ 140 mmHg, DBP of ≥ 90 mmHg, or treatment for previously diagnosed AH; DM was defined as a FPG ≥ 126 mg/dL (7 mmol/L) or use of insulin or other hypoglycemic agents; High TG was defined as a serum triacylglycerol concentration ≥ 200 mg/dL (2.26 mmol/L); Low HDL-C was defined as a HDL-C < 40 mg/dL (1.03 mmol/L) in men and < 50 mg/dL (1.29 mmol/L) in women; The (modified) NCEP MetS was defined as having at least three of the following: (1) WC ≥ 90 cm in men and ≥ 80 cm in women; (2) SBP ≥ 130 mmHg, DBP ≥ 85 mmHg, or self-reported treatment with antihypertensive medications; (3) FPG ≥ 110 mg/dL (6.1 mmol/L) or use of insulin or hypoglycemic agents; (4) serum TG ≥ 150 mg/dL (1.7 mmol/L); (5) low HDL-C

**Figure 1:**
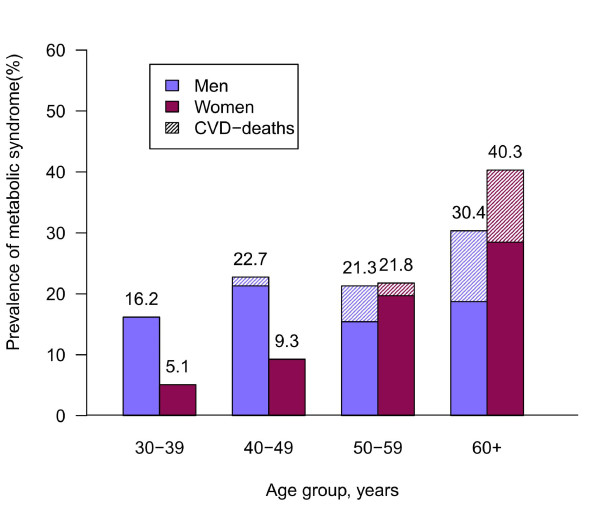
**Prevalence of MetS with age stratification (30-39, 40-49, 50-59, and ≥ 60)**. The shaded areas denote the corresponding proportions of CVD-related deaths. MetS, metabolic syndrome.

During the 32,173 person-years of follow-up, there were 258 all-cause (167 men and 91 women) and 74 CVD-related deaths (42 men and 32 women). Figure 1 shows the prevalence of MetS stratified by age and gender. The MetS prevalence increased with age for both men and women. Men had higher MetS prevalence and CVD deaths than women before age 60. However, the gender difference in prevalence reversed after age 60 (men 30.4%, women 40.3%). The proportions of CVD-related deaths among these MetS subjects had similar trend for both genders, with the death rate increased substantially for women aged over 60.

### Gender and age-specific HRs and PARs

Table 2 lists the gender-specific crude and adjusted HRs and PARs by risk factor for all-cause and CVD-related deaths after controlling for age, BMI, self-reported CAD, stroke, and personal health behaviors such as smoking, alcohol drinking, betel nut chewing, and exercise. For all-cause mortality, AH had the highest HR (95% CI) for both men (1.88, 1.36-2.67) and women (2.19, 1.41-3.88). The association of MetS with all-cause mortality was mediocre in both genders, with HRs of 1.50 (1.03-2.19) in men and 1.77 (1.08-2.89) in women. DM and low HDL-C were also associated with all-cause mortality in men, with HRs of 1.70 (1.12-2.65) and 1.72 (1.22-2.52), respectively. The PARs of these risk factors ranged from 12.6% (4.5-21.8%) for low HDL-C to 26.4% (13.2-38.8%) for AH in men and 29.9% (13.5-47.5%) for AH in women.

**Table 2:** The estimated HRs and PARs of metabolic disorders and MetS for all-cause and CVD-related mortality in men and women

All-cause mortality										
	**Death (%) (N = 167)**	**Crude HR** **(95% CI)**	**Adj HR†** **(95% CI) ‡**	**p-value**	**PAR (%)** **(95% CI) ‡**	**Death (%) (N = 32)**	**Crude HR** **(95% CI)**	**Adj HR†** **(95% CI) ‡**	**p-value**	**PAR (%)** **(95% CI) ‡**

MetS	24 (57.1)	4.74 (2.42-9.28)	3.19 (1.55-7.58)	0.001*	39.2 (15.4-62.5)	20 (62.5)	9.24 (4.59-21.0)	3.45 (1.67-8.83)	0.003*	44.4 (18.7-69.8)

High WC	21 (50.0)	1.98 (1.05-3.69)	1.27 (0.57-2.66)	0.52	10.7 (1.4-40.6)	25 (78.1)	7.14 (3.38-24.3)	3.78 (1.79-13.6)	0.008*	57.5 (29.3-84.3)

AH	32 (76.2)	8.45 (4.19-20.0)	4.07 (1.84-9.98)	0.0005*	57.5 (28.6-79.6)	20 (62.5)	7.33 (3.54-16.4)	2.20 (1.03-5.50)	0.05*	34.1 (6.7-64.5)

DM	16 (38.1)	6.56 (3.29-12.9)	3.39 (1.51-8.61)	0.0004*	26.8 (8.3-48.5)	11 (34.4)	7.81 (3.52-16.6)	2.38 (0.98-5.70)	0.04*	19.9 (3.4-42.3)

High TG	13 (30.9)	1.56 (0.70-2.85)	1.29 (0.55-2.74)	0.47	6.9 (0.5-29.1)	11 (34.4)	4.34 (1.71-9.31)	1.97 (0.77-5.26)	0.10	16.9 (2.1-41.3)

Low HDL-C	15 (35.7)	1.89 (0.92-3.55)	2.07 (0.88-4.63)	0.03*	18.4 (3.0-38.9)	17 (53.1)	3.66 (1.81-7.90)	2.69 (1.25-6.51)	0.008*	33.4 (9.0-59.7)

†: Models were adjusted by age strata, CAD, stroke, BMI category, alcohol drinking, smoking, betel nut chewing, exercise, and income

‡: The 95% confidence interval (CI) obtained by taking the 2.5 and 97.5 percentiles of the recalculated PARs among one thousand bootstrapped datasets

#: CVD-related mortality (ICD-9th revision codes 240-279 and 390-459)

§: N/A: Not available. The adjusted HR was less than 1, yielding a negative corresponding PAR

*: *p*-value < 0.05 for the adjusted HR

BMI, body mass index; CAD, coronary artery disease; CI, confidence interval; CVD, cardiovascular disease; DM, diabetes mellitus; AH, arterial hypertension; HDL-C, high- density lipoprotein cholesterol; HR, hazard ratio; MetS, metabolic syndrome; PAR, population attributable risk; TG, triglycerides; WC, waist circumference

For CVD-related mortality, the respective associations of MetS, DM, and low HDL-C were even stronger, with adjusted HRs of 3.19 (1.55-7.58), 3.39 (1.51-8.61) and 2.07 (0.88-4.63) in men, respectively, and 3.45 (1.67-8.83), 2.38 (0.98-5.70), and 2.69 (1.25-6.51) in women, respectively. The respective associated PARs (%) of the MetS, DM, and low HDL-C were 39.2 (15.4-62.5), 26.8 (8.3-48.5), and 18.4 (3.0-38.9) in men, respectively, and 44.4 (18.7-69.8), 19.9 (3.4-42.3), and 33.4 (9.0-59.7) in women, respectively. While AH had the highest HR (4.07, 1.84-9.98) and PAR (57.5%, 28.6-79.6%) in men, central obesity was not a significant risk factor. In contrast, high WC was the strongest risk factor for women with a HR of 3.78 (1.79-13.6) and a PAR of 57.5% (29.3-84.3%). Of the CVD-related deaths, 8 out of 74 had ICD-9 codes of 415-417, 424-427, 429, and 441, which may not have been directly related to CVD mortality. We re-analyzed our results after excluding these cases and obtained very similar outcomes (data not shown).

**Figure 2:**
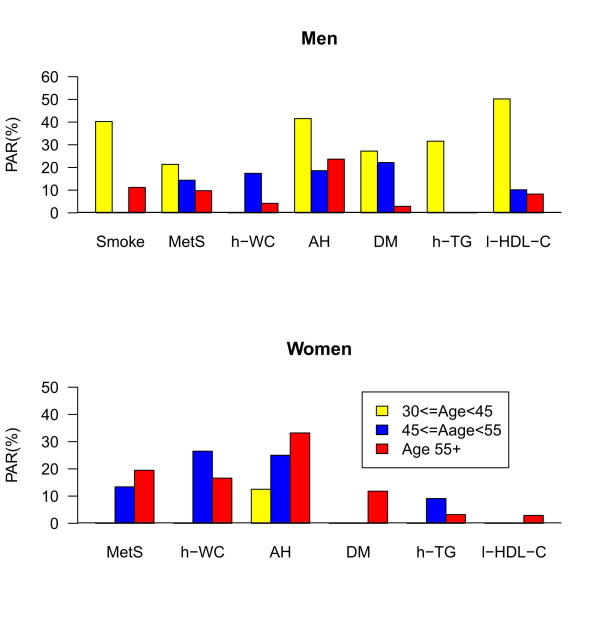
**Metabolic disorder-specific PARs stratified by gender and age groups (smoking for men was included as a risk factor) for all-cause mortality: **a. Men; b. Women. AH, arterial hypertension; DM, diabetes mellitus; h-TG, high triglycerides; h-WC, high waist circumference; l-HDL-C, low high-density lipoprotein cholesterol; MetS, metabolic syndrome; PAR, population attributable risk.

Figure 2 shows the effects of age on the PARs of the risk factors for all-cause mortality. Younger men (30-45 years old) had higher PARs except for high WC. In contrast, older women (≥ 55 years old) had higher PARs than their younger counterparts. The PAR scales of MetS, high WC, and AH were also different in men and women across different age categories. Because smoking was much more prevalent in men than women (53.4% vs. 3.9%), the PAR of smoking was shown as a risk factor in men but not in women. Younger men who smoked had an alarmingly high PAR for all-cause mortality due to cancer (3 out of 3).

**Figure 3:**
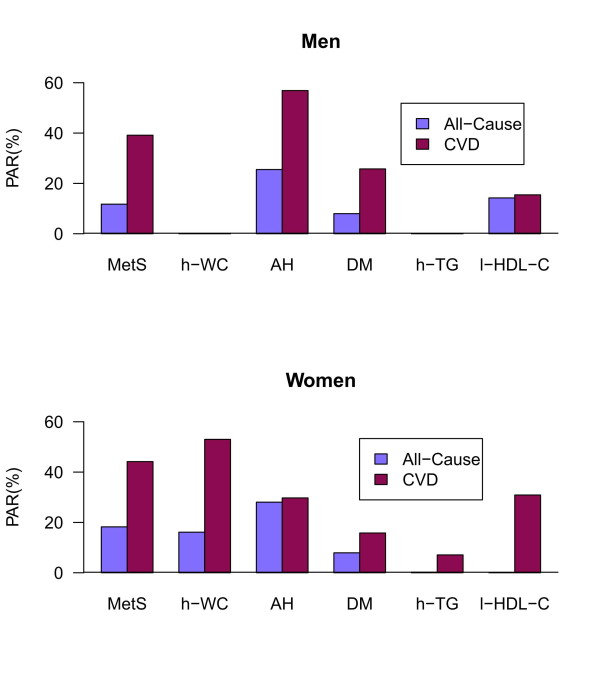
**PARs of MetS and its components after adjusting for the other 4 components**. (The PARs of h-WC and h-TG in men were not shown because of negative values resulting from non-significant adjusted HRs.) AH, arterial hypertension; DM, diabetes mellitus; h-TG, high triglycerides; h-WC, high waist circumference; l-HDL-C, low high-density lipoprotein cholesterol; MetS, metabolic syndrome; PAR, population attributable risk.

The PARs of metabolic disorder for all-cause and CVD-related mortality are shown in Table 2 for the single MetS component without adjusting for other disorders. To assess the effects of the other variables, Figure 3 displays the PARs of MetS and its components after adjusting for the other 4 components, which were quite similar to those in Table 2. The PARs of AH were high in men and women, similar to the levels observed with DM and low HDL-C. However, central obesity had an alarmingly high PAR in women, whereas it was not significant in men.

Table 3 compares the effect of metabolic disorders on all-cause and CVD-related mortality in women according to menopausal status, as determined by peri-menopausal (aged 45-55 years old) or post-menopausal (≥ 55 years old) [25,26]. Because there were very few cases of all-cause and CVD-related mortality for peri-menopausal women (*n *= 15 and 3, respectively), none of the risk factors were significantly associated. However, for post-menopausal women, MetS, AH, and DM were significantly associated with all-cause mortality (HRs 1.89, 2.14, and 1.99, respectively; PARs 19.5%, 33.2%, and 11.8%, respectively). The associations MetS, high WC, DM, and low HDL-C and CVD-related mortality were even stronger in the same age group, with HRs of 2.95, 3.38, 2.78, and 2.51, respectively, and PARs of 42.5%, 47.8%, 22.8%, and 24.5%, respectively.

**Table 3:** Adjusted HRs and PARs of metabolic disorders for all-cause and CVD-related mortality in women stratified by menopausal status (N = 1143)

All-cause mortality						
Peri-menopausal				Post-menopausal		
**45 ≤ Age < 55**				**Age ≥ 55**		

	**Death (%)(n = 15)**	**Adj HR†** **(95% CI) ‡**	**PAR (%)** **(95% CI)‡**	**Death** **(%)(n = 63)**	**Adj HR†** **(95% CI) ‡**	**PAR (%)** **(95% CI)‡**

MetS	4 (26.7)	1.70 (0.33-4.86)	13.4 (1.6-35.3)	35 (55.6)	1.89 (1.05-3.59)*	19.5 (4.1-32.9)

High WC	8 (53.3)	2.33 (0.66-10.90)	26.5 (3.2-51.6)	46 (73.0)	1.48 (0.79-3.44)	16.6 (1.5-39.4)

AH	6 (40.0)	2.48 (0.64-9.38)	25.0 (3.0-47.1)	42 (66.7)	2.14 (1.21-4.27)*	33.2 (11.6-50.6)

DM	0 (0.0)	N/A§	N/A§	22 (34.9)	1.99 (1.02-3.64)*	11.8 (2.18-20.0)

High TG	3 (20.0)	1.64 (0-4.70)	9.1 (0.7-26.7)	17 (27.0)	1.20 (0.64-2.31)	3.2 (0.3-13.5)

Low HDL-C	2 (13.3)	0.61 (0-2.21)	N/A§	21 (33.3)	1.12 (0.58-1.97)	2.9 (0.4-16.2)

CVD-related mortality						

Peri-menopausal				Post-menopausal		

45 ≤ Age < 55				Age ≥ 55		

	Death (%) (n = 3)	Adj HR† (95% CI) ‡	PAR (%) (95% CI)‡	Death (%) (n = 28)	Adj HR† (95% CI) ‡	PAR (%) (95% CI)‡

MetS	2 (66.7)	6.21 (N/A§)	41.9 (4.3-86.8)	18 (64.3)	2.95 (1.39-8.71)*	42.5 (19.4-64.7)

High WC	3 (100)	N/A§	N/A§	22 (78.6)	3.38 (1.52-15.1)*	47.8 (21.2-71.3)

AH	2 (66.7)	6.44 (N/A§)	52.8 (4.0-100)	18 (64.3)	1.93 (0.88-5.50)	36.0 (6.37-68.1)

DM	0	N/A§	N/A§	11 (39.3)	2.78 (1.16-7.31)*	22.8 (4.99-38.6)

High TG	2 (66.7)	11.5 (N/A§)	45.6 (11.2-85.7)	9 (32.1)	1.65 (0.51-4.88)	11.4 (1.4-30.4)

Low HDL-C	1 (33.3)	3.96 (N/A§)	28.0 (2.99-75.0)	15 (53.6)	2.51 (1.06-6.11)*	24.5 (5.22-42.3)

†: CAD, stroke, BMI, alcohol drinking, smoking, betel nut chewing, exercise, and income were adjusted for in the model.

‡: The 95% CI obtained by taking the 2.5 and 97.5 percentiles of the recalculated PARs among one thousand bootstrapped datasets.

§: N/A: not available. The adjusted HR was less than 1 due to small number of observations, yielding a negative corresponding PAR.

#: CVD-related mortality (ICD-9^th ^revision codes 250, 390-398, and 401-441).

BMI, body mass index; CAD, coronary artery disease; CI, confidence interval; CVD, cardiovascular disease; DM, diabetes mellitus; AH, arterial hypertension; HDL-C, high-density lipoprotein cholesterol; HR, hazard ratio; MetS, metabolic syndrome; PAR, population attributable risk; TG, triglycerides; WC, waist circumference

**Figure 4:**
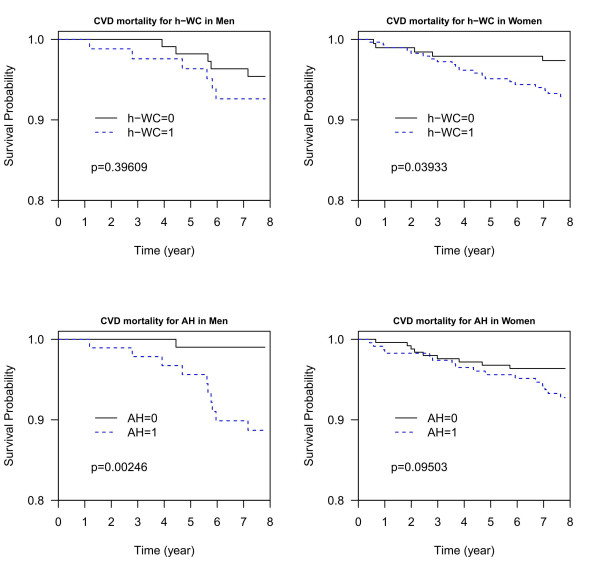
**Kaplan-Meier curves are illustrated for CVD-related morality in subjects 55 years or older both with and without central obesity or hypertension in men (left column) and women (right column)**. a. Central obesity (h-WC = 1, dotted line; h-WC = 0, solid line); b. Arterial hypertension (AH = 1, dotted line; AH = 0, solid line). All the subjects who smoked, drank alcohol, or chewed betel nut were excluded. AH, arterial hypertension; CVD, cardiovascular disease; h-WC, high waist circumference.

To further investigate the gender disparity in central obesity and hypertension, a log-rank test was applied to test for differences between high WC = 1 and 0 (h-WC = 1 and 0) and AH (AH = 1 and 0) for subjects aged ≥ 55 years old who had relatively healthy lifestyles (non-smoking, non-alcohol drinking, and non-betel nut chewing) which included 190 men and 479 women. The Kaplan-Meier curves showed that, while the group difference for high WC was not significant in men (*p *= 0.49), the association was significant in women (*p *= 0.04) (Figure 4a). In contrast, AH was highly significant in men (*p *= 0.003), whereas it was not significant in women (*p *= 0.09) (Figure 4b).

Although smoking is not one of the MetS components, it is an important risk factor for CVD. The HRs of smoking, adjusting for the components of MetS, for all-cause and CVD-related mortalities were 1.27 and 0.89, respectively. When stratified by age (30-45, 45-55, and ≥ 55 years old), the HRs for all-cause mortality in men were 2.16, 0.84, and 1.23, respectively. However, none of the HRs had a significant *p*-value. The HRs of smoking for women is not presented here because of the much smaller smoking rate in women than in men.

## Discussion

Our study provides strong evidence that AH in men and abdominal obesity in women represented the greatest population attributable risks for CVD-related mortality within an 8-year follow-up in Taiwan. The associations between MetS and mortality confirmed the findings of previous studies [6,8,10,11]. However, the attributable risk of each independent MetS component varied with age and gender. Although the prevalence of MetS was higher in men than in women (22.3% vs. 15.8%), the HR was marginally significant and had a smaller PAR for all-cause mortality (11.6% vs. 18.6%). In contrast to the significant associations observed for AH in women, AH, DM, and low HDL-C were all significant in men. The associations were similar for CVD-related mortality, with approximately 40% of the deaths attributable to MetS (for PAR, 39.2% in men and 44.4% in women).

A strong gender disparity was found in our study. AH had the highest PAR in men (57.5%), and high WC had the highest PAR in women (57.5%). Central obesity in men was not significantly associated with mortality. This finding was different from the results of other studies [2,3,7,27]. One possible explanation is that, overall, gender differences for biological susceptibility to atherosclerosis and risky health-related behaviors, including poor food choices, made this disparity significant between men and women [28]. This may be partly due to an increased tendency of left ventricular hypertrophy in men as well as higher smoking rates and alcohol consumption among men [29].

While smoking is not a metabolic disorder, it is the most important risk factor for a coronary event [30]. As shown in Figure 2, younger men aged 30-45 had strikingly high PARs for all-cause mortality. This finding is consistent with studies suggesting that young smokers have higher PARs for coronary heart disease [4,31]. In the Framingham Heart Study, it was found that the competing risk of death from other smoking-related causes shortened median survival by 5 years [32]. A possible link of the elevated PARs of smoking and the metabolic risk factors in younger men were deaths related to cancer or unrecognized cardiac events [33]. The contribution of smoking to the PAR might be expected to operate in part through its probable role in the development of abdominal obesity [34,35]. Therefore, it is somewhat surprising, given the high prevalence of smoking in Taiwanese men, that the PAR is greatest in young men. Perhaps, there are delayed and negated effects of smoking on mortality. This result may provide some insight into why northeast Asian men may achieve relative longevity despite their high rates of cigarette smoking [34-40].

Post-menopausal women are also more prone to central or android obesity, which is more closely associated with the development of type 2 diabetes and increased CVD mortality [14]. This association is most likely due to the decreased levels of circulating estrogen and altered lipid levels after menopause, which increased the risk of mortality over the subsequent 2 years [13,41]. The significant association between MetS and mortality in men was inconsistent with the conclusion of Lin et al. [11], though the association in post-menopausal women was consistent. Furthermore, our results showed that the risk rose more quickly within the first 8 years rather than at a follow-up period of 10 to 15 years. Another consideration is that the assessment of abdominal obesity in northeast Asian may not be the same as in Caucasians [35-37].

In contrast to older women who had higher PARs for MetS and other metabolic risk factors, younger men aged 30-45 also had strikingly high PARs for AH, DM, elevated TG and low HDL-C for all-cause mortality (Figure 2). This result may be due to the "female advantage" in the pre-menopausal women compared to age-matched men for metabolic diseases [14,28]. Younger men with DM or low HDL-C also had higher PARs than the other age groups for CVD-related mortality.

There were some limitations with this study. First, due to the 8-year follow-up period, the number of deaths associated with different risk factors may not have been large enough to yield reliable HR and PAR estimates with the desired 95% CI. However, because the subjects were sampled in a manner proportional to the population size, the causal relationship should be unbiased for the study population. Moreover, for almost all of the significant risk factors, the bootstrapped 95% CIs corresponding to the PAR estimates were within reasonable ranges and had positive lower bounds. Thus, the findings and inferences that were made should be legitimate.

Second, the HRs and PARs of the metabolic disorders given in Tables 2 and 3 were obtained without adjusting for other MetS components. It is not clear what the PARs would be in the presence of a single metabolic disorder or with various combinations of these cardiovascular risk factors. Because of sample size limitations, the current dataset could not provide such information. Further analyses using a larger cohort to assess the corresponding PARs are needed. However, as shown in Figure 3, the PARs of MetS and its components, adjusting for the other 4 components, were quite similar to those shown in Table 2.

Third, we classified the menopausal status of women with a cutoff of 55 years of age, rather than biomarker measurements such as serum FSH (follicle-stimulating hormone, a hormone synthesized and secreted by gonadotrophs of the anterior pituitary gland, which regulates development, growth, pubertal maturation, and reproduction) [11]. Misclassifications may have occurred due to this stratification. The number of peri-menopausal women with CVD-related deaths was very small (n = 3), which may be insufficient to yield any valid statistical inference for this subpopulation. Additionally, there was little information on the rate of use of estrogen or hormone replacement therapy among the study subjects, which may have altered the CVD risk and mortality rates [13].

## Conclusions

In summary, the adjusted PARs show that MetS has a greater relevance for CVD-related mortality than for all-cause mortality over an 8-year follow-up period. However, men with AH and post-menopausal women with central obesity accounted for most of the PAR in CVD-related mortality. Likewise, for all-cause mortality, the highest PARs were for AH in men and women with central obesity. Younger men and older women had higher PARs for all-cause mortality for the various MetS components. The gender differences may reflect the higher prevalence of smoking among men, although we made appropriate adjustments for this variable in our analysis. Focusing on MetS and AH and their associations with all-cause and CVD-related mortality without regard to age or gender may have significant policy implications. Moreover, the PARs of approximately 30% for CVD mortality due to diabetes in men and women and due to low HDL-C in post-menopausal women deserve special attention. However, these recommendations may not be as applicable to MetS or its components when morbidity or non-CVD mortality is considered. Nevertheless, these findings warrant a re-evaluation of the priorities for intervention at the earliest phase in the development of risk factors for premature death.

## Abbreviations

AH: Arterial hypertension; ATP: Adult treatment panel; BMI: Body mass index; CAD: Coronary artery disease; CI: Confidence interval; CVD: Cardiovascular disease; DBP: Diastolic blood pressure; DM: Diabetes mellitus; DOH: Department of Health; FSH: Follicle-stimulating hormone; HDL-C: High-density lipoprotein cholesterol; HR: Hazard ratio; MetS: Metabolic syndrome; NCEP: National cholesterol education program; NHIS: National health interview survey; NTD: New Taiwan dollar; PAR: Population attributable risk; SBP: Systolic blood pressure; TG: Triglycerides; TwHHH: Taiwan survey of hypertension hyperglycemia, and hyperlipidemia; WC: Waist circumference; WHO: World Health Organization.

## Competing interests

The authors declare that they have no competing interests.

## Authors' contributions

WSW, MLW, and CCC were involved in the conception and design of the study and drafted the article. WSW performed the statistical analyses. MLW, CCH, HYC and WCC provided suggestions and conceptual interpretation of the study. All the authors read and approved the final manuscript.

## Pre-publication history

The pre-publication history for this paper can be accessed here:

http://www.biomedcentral.com/1471-2458/12/111/prepub

## References

[B1] Chien KL Hsu HC Sung FC Su TC Chen MF Lee YT Metabolic syndrome as a risk factor for coronary heart disease and stroke: an 11-year prospective cohort in Taiwan community Atherosclerosis 2007 194 214 221 10.1016/j.atherosclerosis.2006.07.033 16979176

[B2] Dhaliwal SS Welborn TA Central obesity and cigarette smoking are key determinants of cardiovascular disease deaths in Australia: a public health perspective Prev Med 2009 49 153 157 10.1016/j.ypmed.2009.07.019 19660494

[B3] Fox KA Despres JP Richard AJ Brette S Deanfield JE Does abdominal obesity have a similar impact on cardiovascular disease and diabetes? A study of 91,246 ambulant patients in 27 European countries Eur Heart J 2009 30 3055 3063 10.1093/eurheartj/ehp371 19778928

[B4] Grau M Subirana I Elosua R Fitó M Covas MI Sala J Masiá R Ramos R Solanas P Cordon F Nieto J Marrugat J Why should population attributable fractions be periodically recalculated? An example from cardiovascular risk estimation in southern Europe Prev Med 2010 51 78 84 10.1016/j.ypmed.2010.03.012 20362610

[B5] Hozawa A Kuriyama S Kakizaki M Ohmori-Matsuda K Ohkubo T Tsuji I Attributable risk fraction of prehypertension on cardiovascular disease mortality in the Japanese population: the Ohsaki Study Am J Hypertens 2009 22 267 272 10.1038/ajh.2008.335 19039309

[B6] Huang KC Lee LT Chen CY Sung PK All-cause and cardiovascular disease mortality increased with metabolic syndrome in Taiwanese Obesity (Silver Spring) 2008 16 684 689 10.1038/oby.2007.112 18239592

[B7] Wong ND Thakral G Franklin SS L'Italien GJ Jacobs MJ Whyte JI Lapuerta P Preventing heart disease by controlling hypertension: impact of hypertensive subtype, stage, age, and sex Am Heart J 2003 145 888 895 10.1016/S0002-8703(02)94787-3 12766749

[B8] Hui SW Liu Z Ho SC Metabolic syndrome and all-cause mortality: a meta-analysis of prospective cohort studies Eur J Epidemiol 2010 25 375 384 10.1007/s10654-010-9459-z 20425137

[B9] Ford ES The metabolic syndrome and mortality from cardiovascular disease and all-causes: findings from the National Health and Nutrition Examination Survey II Mortality Study Atherosclerosis 2004 173 309 314 15064107 10.1016/j.atherosclerosis.2003.12.022

[B10] Hildrum B Mykletun A Dahl AA Midthjell K Metabolic syndrome and risk of mortality in middle-aged versus elderly individuals: the Nord-Trondelag Health Study (HUNT) Diabetologia 2009 52 583 590 10.1007/s00125-009-1271-5 19194692

[B11] Lin JW Caffrey JL Chang MH Lin YS Sex, Menopause, Metabolic Syndrome, and All-Cause and Cause-Specific Mortality-Cohort Analysis from the Third National Health and Nutrition Examination Survey J Clin Endocrinol Metab 2010 95 4258 4267 10.1210/jc.2010-0332 20534759

[B12] Zuo H Shi Z Hu X Wu M Guo Z Hussain A Prevalence of metabolic syndrome and factors associated with its components in Chinese adults Metabolism 2009 58 1102 1108 10.1016/j.metabol.2009.04.008 19481771

[B13] Knopp RH Risk factors for coronary artery disease in women Am J Cardiol 2002 89 28E 34E discussion E-5E 10.1016/S0002-9149(02)02409-8 12084401

[B14] Ren J Kelley RO Cardiac health in women with metabolic syndrome: clinical aspects and pathophysiology Obesity (Silver Spring) 2009 17 1114 1123 10.1038/oby.2009.8 19214173

[B15] Mozaffarian D Kamineni A Prineas RJ Siscovick DS Metabolic syndrome and mortality in older adults: the Cardiovascular Health Study Arch Intern Med 2008 168 969 978 10.1001/archinte.168.9.969 18474761

[B16] Rockhill B Newman B Weinberg C Use and misuse of population attributable fractions Am J Public Health 1998 88 15 19 10.2105/AJPH.88.1.15 9584027 PMC1508384

[B17] Chen CC Wang WS Chang HY Liu JS Chen YJ Heterogeneity of body mass index, waist circumference, and waist-to-hip ratio in predicting obesity-related metabolic disorders for Taiwanese aged 35-64 y Clin Nutr 2009 28 543 548 10.1016/j.clnu.2009.04.017 19473734

[B18] Su TC Bai CH Chang HY You SL Chien KL Chen MF Chen HJ Pan WH Tseng CH Cheng SH Hurng BS Hwang LC Evidence for improved control of hypertension in Taiwan: 1993-2002 J Hypertens 2008 26 600 606 10.1097/HJH.0b013e3282f3b352 18300873

[B19] Wahlqvist ML Chang HY Chen CC Hsu CC Chang WC Wang WS Hsiung CA Is impaired energy regulation the core of the metabolic syndrome in various ethnic groups of the USA and Taiwan? BMC Endocr Disord 2010 10 11 10.1186/1472-6823-10-11 20513248 PMC2891753

[B20] Shih Y-T Hung Y-T Chang H-Y Liu J-P Lin H-S Chang M-C Chang F-C Hsiung CA Wu S-L The design, contents, operation and the characteristics of the respondents of the 2001 National Health Interview Survey in Taiwan T J Public Health 2003 22 6 419 430

[B21] Lu TH Chang HY Hwu CM Chiu HC Yin WH Pan WH Comparison of official coders versus physician panel in assignment of underlying cause of death J Formos Med Assoc 2001 100 365 369 11480244

[B22] Heng D Ma S Lee JJM Tai BC Mak KH Hughes K Chew SK Chia KS Tan CE Tai ES Modification of the NCEP ATP III definitions of the metabolic syndrome for use in Asians identifies individuals at risk of ischemic heart disease Atherosclerosis 2006 186 367 373 10.1016/j.atherosclerosis.2005.07.020 16112123

[B23] Hwang LC Bai CH Chen CJ Prevalence of obesity and metabolic syndrome in Taiwan J Formos Med Assoc 2006 105 626 635 10.1016/S0929-6646(09)60161-3 16935763

[B24] The Definition of Obesity for National Citizens and Principle Guidelines http://www.doh.gov.tw/CHT2006/DM/SEARCH_RESULT.aspx (in Chinese)

[B25] Xu L Tsai KS Kim GS Wu Y Vincendon P Chines AA Constantine GD Efficacy and safety of bazedoxifene in postmenopausal Asian women Osteoporos Int 2011 22 559 565 10.1007/s00198-010-1259-5 20535607

[B26] Wu CY Wu SL Lin SJ Chu CMY Changes in hormone therapy prescriptions among middle-aged women in Taiwan: Implications for health needs at menopause Women's Health Issues 2011 21 153 159 10.1016/j.whi.2010.09.002 21168344

[B27] Bigaard J Tjonneland A Thomsen BL Overvad K Heitmann BL Sorensen TI Waist circumference, BMI, smoking, and mortality in middle-aged men and women Obes Res 2003 11 895 903 10.1038/oby.2003.123 12855760

[B28] Pan WH Hsieh YT Wahlqvist ML Gender-specific roles and needs in food-health security Asia Pac J Clin Nutr 2009 18 642 646 19965359

[B29] Antikainen RL Grodzicki T Beevers DG Webster J Jokelainen JJ Bulpitt CJ Left ventricular hypertrophy by Sokolow-Lyon voltage criterion predicts mortality in overweight hypertensive subjects J Hum Hypertens 2009 23 20 26 10.1038/jhh.2008.102 18754020

[B30] Nilsson PM Nilsson JÅ Berglund G Population-attributable risk of coronary heart disease risk factors during long-term follow-up: the Malmö Preventive Project J Intern Med 2006 260 134 141 10.1111/j.1365-2796.2006.01671.x 16882277

[B31] Schnohr P Jensen JS Scharling H Nordestgaard BG Coronary heart disease risk factors ranked by importance for the individual and community Eur Heart J 2002 23 620 626 10.1053/euhj.2001.2842 11969276

[B32] Lloyd-Jones DM Leip EP Larson MG D'Agostino RB Beiser A Wilson PWF Wolf PA Levy D Prediction of lifetime risk for cardiovascular disease by risk factor burden at 50 years of age Circulation 2006 113 791 798 10.1161/CIRCULATIONAHA.105.548206 16461820

[B33] Borena W Stocks T Jonsson H Strohmaier S Nagel G Bjørge T Manjer J Hallmans G Selmer R Almquist M Häggström C Engeland A Tretli S Concin H Strasak A Stattin P Ulmer H Serum triglycerides and cancer risk in the metabolic syndrome and cancer (Me-Can) collaborative study Cancer Causes Control 2011 22 291 299 10.1007/s10552-010-9697-0 21140204

[B34] Shimokata H Muller DC Andres R Studies in the distribution of body fat III Effects of cigarette smoking JAMA 1989 261 1169 1173 2915440

[B35] Matsushita Y Nakagawa T Yamamoto S Takahashi Y Noda M Mizoue T Associations of smoking cessation with visceral fat area and prevalence of metabolic syndrome in men: the Hitachi health study Obesity (Silver Spring) 2011 19 647 651 10.1038/oby.2010.237 20966912

[B36] Nakamura KNH Hara M Higaki Y Imaizumi T Taguchi N Sakamoto T Horita M Shincni K Tanaka K Optimal cutoff values of waist circumference and the discriminatory performance of other anthropometric indices to detect the clustering of cardiovascular risk for metabolic syndrome in Japanese men and women Environ Health Prev Med 2011 16 52 60 10.1007/s12199-010-0165-y 21432217 PMC2999681

[B37] Oka R Kobayashi J Yagi K Tanii H Miyamoto S Asano A Hagishita T Mori M Moriuchi T Kobayashi M Katsuda S Kawashiri M Nohara A Takeda Y Mabuchi H Yamagishi M Reassessment of the cutoff values of waist circumference and visceral fat area for identifying Japanese subjects at risk for the metabolic syndrome Diabetes Res Clin Pract 2008 79 474 481 10.1016/j.diabres.2007.10.016 18031862

[B38] Seidell JC Environmental influences on regional fat distribution Int J Obes 1991 15 Suppl 2 31 35 1794935

[B39] Seidell JC Bjorntorp P Sjostrom L Abdominal obesity and metabolism in men-possible role of behavioural characterisitcs Obesity in Europe 1989 88 91 97

[B40] Seidell JC Bjorntorp P Sjostrom L Kvist H Sannerstedt R Visceral fat accumulation in men is positively associated with insulin, glucose, and C-peptide levels, but negatively with testosterone levels Metabolism 1990 39 897 901 10.1016/0026-0495(90)90297-P 2202881

[B41] Silva TC Barrett-Connor E Ramires JA Mansur AP Obesity, estrone, and coronary artery disease in postmenopausal women Maturitas 2008 59 242 248 10.1016/j.maturitas.2008.01.008 18374526

